# Photobiomodulation associated with physical exercise in shoulder impingement syndrome. Systematic review with meta‐analysis

**DOI:** 10.1111/php.14113

**Published:** 2025-05-14

**Authors:** Giulia de Lara Quagliotto, Milena Pastor Manchope, Rebeca Hilario, Vitoria Zubeldia, Renata Anddresa Stacheslki, Alberito Rodrigo de Carvalho, Márcia Rosângela Buzanello, Gladson Ricardo Flor Bertolini

**Affiliations:** ^1^ Universidade Estadual do Oeste do Paraná – Unioeste Cascavel Brazil

**Keywords:** exercise, low‐level light therapy, shoulder pain

## Abstract

To present the use of PBM associated with physical exercise in the treatment of shoulder impingement syndrome, with its different parameters. The following databases were used: PubMed, Web of Science, Embase, Cochrane, Scopus, and Lilacs. The gray literature included Google Scholar, Open Grey, LIVIVO, and the Brazilian Library of Theses and Dissertations. Ten randomized clinical trials were included. Pain intensity was improved with PBM compared to control [MD = −0.89, 95% CI (−1.38, −0.40), *I* ^2^ 46%, *p* = 0.0004]. The 3 different times of assessment (at rest, activity, and at night) of pain intensity were not statistically significant and likewise, the combined effect size [SMD = −0.16; 95% CI (−0.43, 0.12), *I* ^2^ 63%, *p* = 0.26]. A significant improvement in ROM was seen in the PBM group compared to the control [MD = 12.24, 95% CI (7.64, 16.84), *I* ^2^ 85%, *p* < 0.00001]. The combined effect estimate showed no significant improvement in functionality in the PBM group compared to the control [MD = −1.47, 95% CI (−7.34, 4.41), *I* ^2^ 58%, *p* = 0.62]. PBM therapy combined with physical exercise is effective in reducing pain and improving the range of motion in individuals with shoulder impingement syndrome.

## Graphical abstract

The combination of physical exercise and PBM therapy has been demonstrated to be an effective approach in reducing pain and enhancing range of motion and functionality.

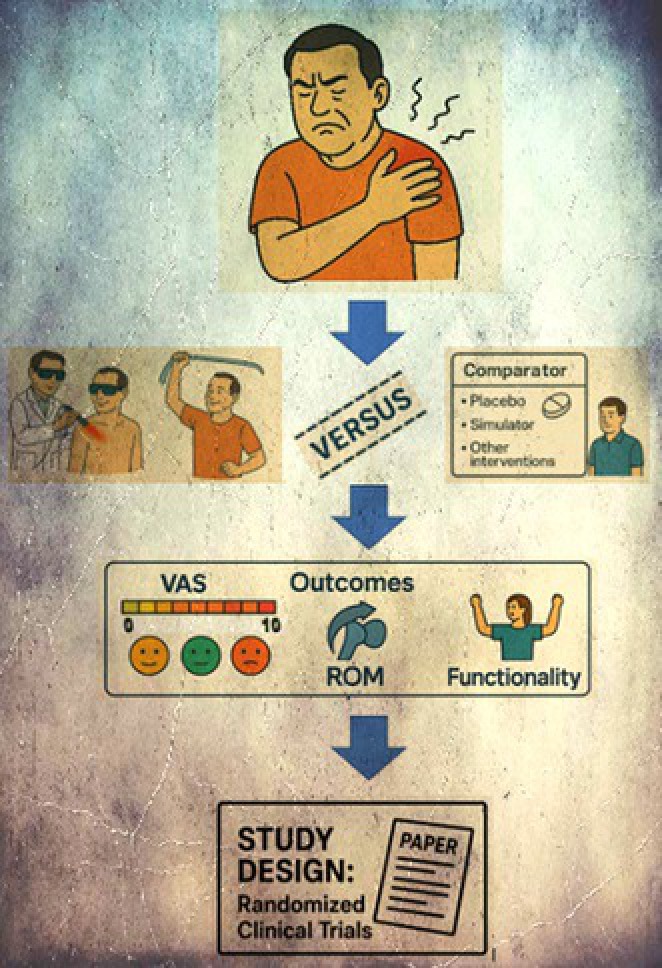

**Abbreviations**
DASH: Disability of arm, shoulder, and hand
DeCS: descriptors defined in Health Sciences
MeSH: Medical Subject Headings
NPRS: Numeric Pain Rating Scale
PBM: photobiomodulation
PRISMA: Preferred Reporting Items for Systematic Reviews and Meta‐Analyses
QCRI: Qatar Computing Research Institute
ROM: Range of motion
SDQ: Shoulder Disability Questionnaire
SIS: shoulder impingement syndrome
SPADI: Shoulder pain and disability index
UCLA: University of California Los Angeles end result score
VAS: Visual Analog Scale
VHL: Virtual Health Library

## INTRODUCTION

Shoulder pain is a very common musculoskeletal disorder and can be disabling.^1^ It can arise from athletes to sedentary individuals who perform intense activities with their upper limbs, especially when associated with psychological problems, continuous use of computers, and uncomfortable postures, among other factors.^2^ Among the diseases affecting this joint, the most common is shoulder impingement syndrome (SIS), which is characterized by a chronic inflammatory process generated by compression of the rotator cuff tendons, especially the supraspinatus portion, and the subdeltoid pouch against the coracoacromial arch, during shoulder movements above the head line. SIS can be primary, when there is a reduction in the subacromial space, or secondary, when there is a functional disorder.^3^, ^4^

Treatment for shoulder impingement syndrome can be surgical^5^ or conservative, which includes cessation of painful movements, anti‐inflammatory medication, corticosteroid injections, and physiotherapy.^4^, ^6^ However, due to the side effects that occur with drug treatment,^7^, ^8^ physiotherapeutic treatment stands out, with a wide range of protocols including physical exercise, manual therapy, electrothermotherapy, and photobiomodulation (PBM), although there is controversy about the use of the latter.^9^, ^10^, ^11^, ^12^, ^13^, ^14^, ^15^

The general therapeutic effects of PBM are modulation of the inflammatory process, aid in tissue regeneration, and pain relief.^16^, ^17^, ^18^, ^19^ However, the clinical efficacy of low‐intensity laser therapy in the treatment of SIS remains controversial.^13^, ^20^, ^21^, ^22^, ^23^ However, the discrepancies between the studies may be due to the use of different protocols, due to the different dosimetric parameters.^24^ Thus, the aim of this systematic review is to present the use of PBM associated with physical exercise in the treatment of shoulder impingement syndrome, with its different parameters.

## MATERIALS AND METHODS

### Protocol

This systematic review was conducted and reported in accordance with Preferred Reporting Items for Systematic Reviews and Meta‐Analyses (PRISMA). Registered with the Open Science Framework DOI 10.17605/OSF.IO/HQCRP.

### Eligibility criteria

The acronym PICOS was used to formulate the question of this study: P—population: individuals with shoulder impingement syndrome; I—intervention: low‐intensity laser therapy with possible association with physical exercise; C—comparison: control group, placebo, or other interventions; O—outcomes: pain (primary outcome), range of motion, and functionality (secondary outcomes); S—study design: randomized clinical trials.

Inclusion criteria: men and women diagnosed with shoulder impingement syndrome. Individuals with painful and limited passive glenohumeral mobility; more restricted lateral rotation compared to abduction and medial rotation; and no clear signs that the shoulder pain was caused by another condition.

Exclusion criteria: individuals diagnosed with insulin‐dependent diabetes mellitus; systemic inflammatory joint disease; treatment with corticosteroids or physiotherapy in the last 3 months; severe infection; uncontrolled hypertension; shoulder surgery, dislocation, or fracture; calcification over the shoulder joint; pregnancy; complete rupture of the rotator cuff; presence of direct trauma to the shoulder, acromioclavicular arthritis; presence of underlying neurological disease or extrinsic diseases; study designs other than randomized clinical trials.

### Sources of information

The initial search was carried out using keywords in the PubMed database, with the Medical Subject Headings (MeSH) medical metadata system, descriptors defined in Health Sciences (DeCS) from the Virtual Health Library (VHL) website, and also free terms. Individual search strategies were developed for the following databases: PubMed, Web of Science, Embase, Cochrane, Scopus, and Lilacs. There were also searches in gray literature: Google Scholar, Open Grey, LIVIVO, and the Brazilian Library of Theses and Dissertations. There were no limitations as to period or language. The preliminary searches in all the databases were carried out on February 13, 2024, and updated on June 11, 2024, totaling 843 references (Appendix S1).

### Study selection and data collection process

The study selection process was carried out using two reference managers, EndNote Web and Rayyan – Qatar Computing Research Institute (QCRI). The articles were imported from the databases into the Endnote Web reference manager for automatic and manual removal of duplicate articles. They were then imported into Rayyan and, once again, duplicates were removed manually by the first reviewer. The studies included in Phase 1 (reading of titles and abstracts) were then defined according to the eligibility criteria by two blinded reviewers (R1 and R2), followed by selection for Phase 2 (reading of full texts). Studies with conflicts were resolved by a third reviewer (R3).

### Data collected

Data were collected on the characteristics of the studies (authors, year of publication, country), the sample (sample size, mean age, and gender), interventions, outcome assessment instruments, outcomes, assessment/follow‐up periods, and conclusions.

### Individual assessment of the risk of bias in studies

The risk of bias was assessed by blinded reviewers R1 and R2, using the Cochrane tool RoB2, with any discrepancies resolved by R3. All the included studies were assessed in five domains: bias in the randomization process; deviations from the intended intervention; bias due to missing data; bias in the measurement of outcomes; and bias in the selection of reported results, in addition to the overall risk of bias. Each domain had an overall result: low risk, some concern, or high risk.

### Meta‐analysis

Statistical analysis was carried out using RevMan 5.4.1 (The Cochrane Collaboration, Software Update, Oxford, UK). Continuous results were expressed as standardized mean differences and mean differences with ninety‐five percent confidence intervals (95% CI). A *p*‐value <0.05 was considered statistically significant. The value of the *I* ^2^ statistical test was calculated to test for heterogeneity between studies. A value of *I* ^2^ ≥ 50% was considered significant heterogeneity. A random effects model was adopted. When necessary, transformations were used according to the Cochrane Handbook.^24^

### Risk of publication bias

To minimize the likelihood of publication bias, a comprehensive study was carried out, with no language or publication period restrictions. The gray literature and reference list of the studies were also included. In this way, the possibility of publication bias was reduced, although not completely eliminated. Analysis of the symmetry of the funnel plot was also carried out.

## RESULTS

The following is a narrative synthesis and meta‐analysis of the included studies, structured around the reported results.

### Selection of studies

**FIGURE 1:**
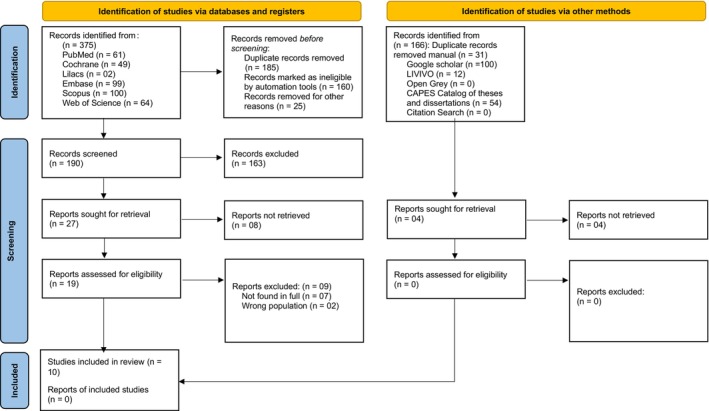
PRISMA 2020 flow diagram for new systematic reviews which included searches of databases, registers, and other sources.

During the search, 541 studies were found, 375 in the main databases and 166 in the gray literature. The search was carried out on February 13, 2024, and updated on June 11, 2024 (Appendix S1). Of the total number of studies, 160 duplicates were automatically excluded from the indexed databases, and 25 were manually excluded from the indexed databases, and 31 from the gray literature. This left 325 studies for Phase 1 (reading titles and abstracts) and 19 for Phase 2 (reading full texts). After screening, 10 studies were included in this review (Figure 1).

### Individual characteristics of the studies

Table 1 summarizes the main characteristics of the ten randomized clinical trials that were included in this review. Publication dates ranged from 2009 to 2023. The total number of individuals sampled was 523, including both men and women. Most of the studies were conducted in Turkey.^21^, ^22^, ^23^, ^25^, ^26^ The others were completed in Iran,^27^, ^28^ one in Egypt,^29^ one in India,^30^ and one in Brazil.^31^

**TABLE 1:** Summary characteristics of the clinical trials included in this review (*n* = 10).

Author/year	Country	Sample characterization	Study design	Intervention and control groups	Assessment and scales	Conclusion
Bal et al. (2009)	Turkey	*N* = 40 EG: (*n* = 20) CG: (*n* = 20) Age: EG: 51.7 ± 14.1 CG: 53.1 ± 8.4 Gender: EG: F (15), M (5) CG: F (13), M (7)	Prospective randomized clinical trial	EG: LLLT + exercise + hot pack + cold pack CG: Exercise + hot pack + cold pack	T0: baseline T1: 2nd week T2: 12th week Night pain severity: VAS (0–100 mm) Functional status: SPADI t, p, d Effectiveness of treatment UCLA*: 35 points (Poor, Good and Excellent)	The study failed to show a distinct advantage of LLLT over exercise alone. A comprehensive home exercise program should be the main therapeutic option for the rehabilitation of SIS
Yeldan et al. (2009)	Turkey	*N* = 60 EG: (*n* = 34) PG: (*n* = 26) Gender: EG: F (25), M (9) PG: F (22), M (4) Age: EG: 55.32 PG: 55.00	Prospective randomized clinical trial	EG: LLLT + exercise + cold CG: Placebo laser + exercise + cold	T0: baseline T1: 3rd week No follow‐up Pain severity: VAS: (0–10 cm) VAS A (activity) VAS R (rest) VAS N (at night) Functional assessment: Constant Scoring System – 100 Shoulder disability: DASH SDQ ROM (degrees): Goniometer	There is no fundamental difference between LLLT and placebo LLLT when they are supplementing an exercise program for rehabilitation of patients with SIS
Abrisham et al. (2011)	Iran	*N* = 80 EG: (*n* = 40) PG: (*n* = 40) Age: EG: 52.2 ± 5.7 PG: 51.2 ± 6.7 Gender: EG: F (24), M (16) PG: F (26), M (14)	Randomized, double‐blind, controlled trial	EG: LLLT + exercise CG: Placebo laser + exercise	T0: baseline T1: post‐treatment, 2nd week No follow‐up Pain severity: VAS: 0–10 cm ROM (degrees): Goniometer active and passive: (flexion, abduction, adduction, external rotation)	LLLT combined with exercise therapy is more effective than exercise therapy alone in relieving pain and improving shoulder joint ROM in patients with SIS
Dogan et al. (2010)	Turkey	*N* = 52 EG: (*n* = 30) PG: (*n* = 22) Age: EG: 53.7 ± 12.6 CG: 53.45 ± 9.64 Gender: EG: F (20), M (10) PG: F (13), M (9)	Randomized, placebo controlled, double‐blind prospective	EG: LLLT + exercise + cold PG: Placebo laser + exercise + cold	T0: baseline T1: post‐treatment, 14th day No follow‐up Pain severity: VAS: (0–10 cm) ROM (degrees): Goniometer (flexion, abduction, adduction, external rotation) Functional status: SPADI p, d, t	There were improvements in pain severity, ROM and functional status of SIS patients with LLLT, cold pack and exercise. However, no superiority over placebo laser therapy was observed
Eslamian et al. (2011)	Iran	*N* = 50 EG: (*n* = 25) CG: (*n* = 25) Age: EG: 50.16 ± 12.10 PG: 50.28 ± 11.72 Gender: EG: F (10), M (15) CG: F (16), M (9)	Randomized double‐blind controlled trial	EG: LLLT + exercise + hot pack + US + TENS CG: Exercise + hot pack + US + TENS	T0: baseline T1: follow‐up – 3rd weeks after termination of treatment Pain severity: VAS (0–10 cm) ROM (degrees): Goniometry (active and passive: abduction and external rotation) Functional status: SDQ	The combination of LLLT with conventional physiotherapy is superior to routine physiotherapy from the point of view of reducing pain and improving patient function, but not in increasing ROM compared to other physical agents
Somashekhar et al. (2014)	India	*N* = 60 EG: (*n* = 30) CG: (*n* = 30) Age: EG: 27.60 ± 1.72 PG: 28.13 ± 1.41 Gender: EG: F (13), M (17) CG: F (15), M (15)	Random sampling	EG: Laser + exercise CG: US + exercise	T0: baseline T1:3rd day T2: 6th day T3: 9th day T4: 12th day T5: 15th day No follow‐up Pain severity: VAS (0–10) ROM (degrees): Goniometry	ROM and pain had significant improvements in EG compared to CG
Abd‐Allah et al. (2017)	Egypt	*N* = 30 EG: (*n* = 15) CG: (*n* = 15) Age: EG: 42.46 ± 6.13 CG: 41.46 ± 6.97 Gender: EG: F (8), M (7) CG: F (11), M (4)	Randomized controlled trial	EG: LLLT + Exercise CG: Exercise	T0: baseline T1: post‐treatment, 12th day No follow‐up Pain severity: VAS (0–10 cm)	Eccentric exercise is as effective as LLLT in relieving pain in patients with SIS when added to scapular and rotator cuff muscle strengthening and flexibility exercises
Calis et al. (2011)	Turkey	*N* = 31 EG: (*n* = 15) CG: (*n* = 16) Age: EG: 46.2 ± 12.14 CG: 50.34 ± 13.69 Gender: EG: F (10), M (5) CG: F (11), M (5)	Randomized controlled trial	EG: LLLT + exercise + hot pack CG: Hot pack + exercise	T0: baseline T1: 15th day No follow‐up Pain severity: (0–10) VAS R VAS A VAS N ROM (degrees): Goniometry (abduction, flexion, internal and external rotation) Functional assessment: Constant t	Exercise may be sufficient for short term the treatment of SIS
Alfredo et al. (2020)	Brazil	*N* = 84 EG: (*n* = 42) CG: (*n* = 42) Age: EG: 51.9 ± 8.7 CG: 56.0 ± 10.4	Randomized controlled trial	EG: LLLT + Exercise CG: Exercise	T0: baseline T1: 8 weeks Follow‐up: 3 months (after treatment end) Pain intensity (0–10): NPRS A R ROM (degrees): Goniometry Active (flexion, extension, abduction, adduction, external and internal rotation) Functional status: SPADI: f, p, t Shoulder function: UCLA**	LLLT therapy combined with exercise reduces pain intensity, improves shoulder function and reduces the use of medication over 3 months
Sen et al. (2023)	Turkey	*N* = 36 EG: *n* = 19 CG: *n* = 17 Age: EG: 52.2 ± 8.3 CG: 54.7 ± 10.7 Gender: EG: F (15), M (4) CG: F (13), M (4)	Single‐blind, prospective, randomized controlled	EG: LLLT + cold pack + Exercise CG: Cold pack + Exercise	T0: baseline T1: 1 month Follow‐up: 3 months Pain severity: (0–10) VAS R VAS A VAS N ROM (degrees): (flexion, abduction, internal rotation, and external rotation) Functional status: SPADI: f, p, t	LLLT combined with exercise is more effective in relieving pain and improving functionality than exercise alone in the short term. However, LLLT and US do not provide additional effects in terms of pain and disability at 3 months

*Note*: *p < 0.05.

Abbreviations: A, activity; CG, Control group; DASH, Disability of arm, shoulder, and hand; EG, Experimental group; F, Female; f, function; HAQ, Health‐assessment questionnaire; M, Male; N, night; NPRS, Numeric pain rating scale; p, pain; R, rest; ROM, Range of motion; SDQ, Disability Questionnaire; SIS, Subacromial impingement syndrome; SPADI, Shoulder pain and disability index; t, total; UCLA**, Modified‐University of California at Los Angeles Shoulder Rating Scale; UCLA*, University of California–Los Angeles end result; VAS, Visual analog scale.

Six studies referred to the use of or no use of medication during the trial. Oral paracetamol (1500 mg/d) was used when necessary in the study of Bal et al.,^22^ similar to the use of paracetamol in the study by Calis et al. study et al.^25^ In the study by Alfredo et al.,^31^ the participants were instructed not to use analgesic medication, apart from paracetamol (500 mg/day) or anti‐inflammatory drugs during the study. Abrisham et al.^27^ did not allow painkillers and/or NSAIDs. In the Eslamian trial et al.,^28^ patients were not asked to continue or stop taking NSAIDS. And finally, acetaminophen was recommended to subjects as needed in Sen et al. study.^26^

### Measuring instruments

#### Pain intensity – primary outcome

Pain intensity was assessed in all the studies included in this review. In both pain scales, the visual analog scale (VAS) and the Numeric Pain Rating Scale (NPRS), the higher the score, the greater the pain. Nine studies used the VAS, with a scale of 0–10^21^, ^23^, ^25^, ^26^, ^27^, ^28^, ^29^, ^30^ and 0–100.^22^ Alfredo et al.^31^ used the NPRS with a scale of 0–10.

#### Range of motion – secondary endpoint

Range of motion was assessed in eight studies using goniometry.^21^, ^23^, ^25^, ^26^, ^27^, ^28^, ^30^, ^31^ Only three studies reported whether the movement was active or passive.^27^, ^28^, ^31^ The study by Sen et al.^26^ did not report ROM results.

#### Functionality and disability – secondary outcome

##### Shoulder pain and disability index (SPADI)

Four studies^21^, ^22^, ^26^, ^31^ used the SPADI, which is a quality‐of‐life questionnaire developed to assess pain and disability associated with shoulder dysfunction. The higher the score, the worse the shoulder dysfunction condition.

##### Shoulder Disability Questionnaire (SDQ)

The studies by Yeldan et al.^23^ and Eslamian et al.^28^ used the SDQ questionnaire. The higher the score, the worse the functional status limitation.

##### Disability of arm, shoulder, and hand (DASH)

Yeldan et al.^23^ used the DASH to assess disability. The higher the score, the greater the disability.

##### University of California–Los Angeles end result score (UCLA) e Modified–University of California at Los Angeles (Modified‐UCLA) Shoulder Rating Scale

This instrument was used by Bal et al.^22^ and Alfredo et al.^31^ When interpreting the final UCLA score, the higher the score, the better the shoulder function.

##### Constant scoring system

The studies by Yeldan et al.^23^ and Calis et al.^25^ used this system. The higher the score, the better the functional status.

#### Interventions

The interventions in the ten studies varied considerably, as did the comparators. In two studies, PBM + exercise + cold was compared to Placebo laser + exercise + cold.^21^, ^23^ Similarly, two other studies^29^, ^31^ used PBM + exercise + cold compared to Exercise + cold. The other six studies had different interventions and comparators as follows. One study used PBM + exercise + hot pack + cold compared to Exercise + hot pack + cold.^22^ PBM + exercise was compared to Placebo laser + exercise by Abrisham et al.^27^ Calis et al.^25^ used PBM + exercise + hot pack compared to Exercise + hot pack. Eslamian et al.^28^ compared PBM + exercise + hot pack + US + TENS to Exercise + Hot pack + US + TENS. Finally, Somashekhar et al.^30^ used PBM + exercise compared to US + exercise. More details on dosimetry and treatment frequency are available in Table 2.

**TABLE 2:** Description of protocols and frequency of interventions.

Eligible studies	Intervention protocol
Bal et al. (2009)	EG: LLLT + exercise + hot pack + cold pack CG: Exercise + hot pack + cold pack LLLT: Ga‐As, 904 nm, 5500 Hz frequency, 27 W maximum power output per pulse, 13.2 mW average power, 0.8‐cm^2^ spot size, 1.6 J of total energy was delivered per point at each session at a power density of 16.5 mW/cm^2^, and the cumulative energy per point for all sessions was 16 J LLLT: 5× week/for 10 min each time/for 2 weeks/ total 10 sessions Target location: on the greater and lesser tubercles, the anterior and posterior faces of the capsule and the subacromial regions Exercise: comprehensive home exercise program for 12 weeks Initially, pendulum circumduction and passive shoulder self‐stretching were prescribed. The exercise protocol was followed in order by isometrics in all planes; theraband exercises with three different therabands (low, medium, and high resistances); strengthening exercises for the muscles of scapular stabilization; and advanced muscle strengthening exercises with dumbbells. All the patients were invited to the clinic twice weekly to ensure compliance and to be instructed regarding the new exercise Hot pack application before and cold pack application after shoulder exercises
Yeldan et al. (2009)	EG: LLLT + exercise + cold CG: Placebo laser + exercise + cold LLLT: GaAs diode, 904 nm, 90 s at each location, 2000 Hz, 5–7000 Hz and maximum peak power of 27 50 or 2764 W, each session consisted of three pulses (3 J) to each of a maximum of five tender points, 8 min each session Target location: superior and anterior periarticular parts of glenohumeral joint, covering an area of 15 cm^2^ Exercise: subjects followed a progressive exercise program that included range of motion (ROM), strengthening, and stretching exercises, performed twice daily under supervision in the clinic and at home. In the first week, exercises included inferior and posterior capsule stretching, wand exercises (shoulder flexion, abduction, extension, internal, and external rotation), active‐assisted ROM exercises, and internal rotator exercises using a towel. In the later weeks, these exercises were performed actively with theraband resistance. Additionally, supraspinatus exercises (empty can) were introduced in the second and third weeks Exercise: each session was a minimum of 15 min and a maximum of 30 min Superficial cold and progressive exercise: 5 days/week, for 3 weeks At the end of the therapy, cold pack was applied around the shoulder for 15 min
Dogan et al. (2010)	EG: LLLT + exercise + cold PG: Placebo laser + exercise + cold LLLT: 850 nm, power output of 100 mV, continuous wave and 0.07 cm^2^ spot area laser were used for the laser therapy. The laser was applied with a dosage of 5 joule/cm^2^ (totally 15–20 joule) at maximum 5–6 painful points for 1 min at each point (3 joule/cm^2^ at each point over maximum 5–6 painful points for 1 min) Target location: over subacromial region of the shoulder Cold pack: 10 min Exercise: range of motion, stretching and progressive resistive exercises. Each exercise was performed once a day with 10–15 repetitions 5×/week, once a day for 14 sessions
Abrisham et al. (2011)	EG: LLLT + exercise CG: Placebo laser + exercise LLLT: 890 nm, in pulsed mode, 2–4 J/cm^2^ in each three points. In each treatment session, three points on the shoulder were irradiated for 2 min (total of 6 min) Target location: (coracoid), posterior (glenohumeral joint), and lateral (rotator cuff tendon). Also, the biceps tendon was irradiated for patients with biceps tendinitis Exercises: strengthening, stretching, and mobilization exercises in clinic and at home Exercise in clinic: pulley and shoulder wheel exercises in all the sessions Home exercise: pendular shoulder exercise for two first sessions. From the third session, isometric shoulder exercises and active‐assisted exercise (deltoid, biceps, triceps, and scapula fixator muscles) Laser after the exercise therapy programs 5 days/week, for 2 weeks
Calis et al. (2011)	EG: LLLT + exercise + hot pack CG: Hot pack + exercise Hot pack: moist hot pack, 20 min LLLT: 904 nm, 6 mW, 1 J/cm^2^, 16 Hz, 2 min duration Target location: direct contact with 90° straight to the shoulder Exercise: initially—passive mobilization and Codman exercises. Subsequently—stretching and strengthening rotator cuff, biceps brachii, deltoid, and other shoulder muscles Exercise: 5×/5 s each session, 5 days a week, for 15 days, same physiotherapist US and LLLT was applied for 15 days
Eslamian et al. (2011)	EG: LLLT + exercise + hot pack + US + TENS CG: Exercise + hot pack + US + TENS LLLT: Ga‐Al‐As, 830 nm, average power output of 100 mW, and energy density or intensity of 4 J/cm^2^. Continuous wave mode on 1‐cm^2^ surface area with 20‐s irradiation for each point and total treatment duration of 5 min Target location: over the painful regions of shoulder up to ten painful points Hot pack: underwent superficial heat therapy US: pulse mode, 1‐MH^z^ frequency, pulse intensity: 1.5–2 W/cm^2^ and duty factor: 25% for 5‐min treatment duration with slowly circular movements of ultrasound probe over painful regions of shoulder TENS: 100 Hz, low current intensity of 10–30 mA, and short pulse width or 50 μs, for 20 min Exercise: Included ROM, stretching and strengthening for the abductors and flexors of the shoulder. 1×/day of 10 repetitions, 3×/week/total 10 sessions
Somashekhar et al. (2014)	EG: LLLT + exercise CG: US + exercise LLLT: 850 nm, power output 100 mV, dosage 5 J/cm^2^, beam area 0.07 cm^2^, duration: 5–6 min US: Intensity 0.5–0.8 W/cm^2^, pulsed mode, coupling media—ultrasonic gel, transducer size – 1 cm^2^, frequency 3 MHz, technique—direct contact in small concentric circles, treatment time 3–4 min Target location: not reported Exercise: Codman's pendular exercise. Shoulder external rotation, internal rotation, and abduction using Theraband: Theraband shoulder external rotation at 0° (starting at 45°), Theraband shoulder abduction to 45°, Theraband shoulder Internal rotation at 0° (starting at 45°) 5×/week/for 2 weeks/total 10 sessions
Abd‐Allah et al. (2017)	EG: LLLT + Exercise CG: Exercise LLLT: 905 nm, 3 MHz pulsed mode, 4 points (3 points anteriorly and 1 point laterally), with a distance about 1 cm between each point measured with a ruler, 150 s for each point Target location: patient shoulder behind the patient back Flexibility and strengthening exercises: The treatment involved stretching the posterior shoulder capsule, performed 3 times at the beginning of the session, with a 30‐s hold and 10 s of rest between repetitions. Strengthening exercises using a Thera‐band included serratus anterior strength, shoulder scaption, external rotation, and scapular retraction. Three sets of 10 repetitions were completed for each exercise, with a 1‐min rest between sets, and the resistance level was adjusted as needed. Patients were instructed to perform the exercises in the maximal range of motion they could achieve with minimal shoulder pain. Resistance was increased as tolerated using bands of different levels (orange, red, and blue). Eccentric training involved slowly lowering the arm from the starting position, with the load gradually increased as pain decreased. This included 3 sets of 15 repetitions with a 1‐min rest between sets, and weight was progressively increased by 1/2 kg as long as the pain was tolerable (not exceeding 5 on a 0–10 pain scale) 3 sessions per week, for 4 weeks
Alfredo et al. (2020)	EG: LLLT + Exercise CG: Exercise LLLT: gallium arsenide diode, 904 nm, 700 Hz, average power 60 mW, peak pulse power of 20 W, and 50 s of irradiation per point (area, 0.5 cm^2^). 3 J point, total dose 27 J per treatment Target location: over 3 insertion points each in the supraspinatus muscle tendon, on the subacromial bursa, and along the bicipital groove Supervised exercises: Isotonic strengthening: scapular pivot, scapula stabilizer, and humeral propellant muscle groups. 3 sets of 15 repetitions of each Isometric exercises: 10 sets of 10 s each Stretching: each muscle group was performed for 30 s being performed 3 times in a row The progression of the exercises occurred in three stages: First stage (first six sessions): 10 sets of 10 s each; Second stage (next nine sessions): 3 sets of 15 repetitions with a 1‐kg load; Third stage (last nine sessions): 3 sets of 15 repetitions with a 1.5 kg load 3×/week, for 8 weeks
Sen et al. (2023)	EG: LLLT + cold pack + Exercise CG: Cold pack + Exercise LLLT: gallium aluminum‐arsenide diode, 850 nm, 100 mV, 3 J/cm^2^, continuous wave, 5 points, for 1 min at each point Target location: over the greater and lesser tubercles, the bicipital groove, and the anterior and posterior aspects of the capsule Exercise: home‐based exercise (HBE) program: posture, pectoral and trapezius exercises, shoulder ROM stretching exercises. Codman and ladder exercises with the fingers. After reaching full active ROM, they performed strengthening exercises for the rotator cuff and scapula. The intensity of the exercise was gradually increased and the number of sets LLLT: 5 sessions/week, 1/day, total of 15 sessions for 3 weeks Cold pack therapy: applied for 10 min Exercise: home‐based exercise (HBE) program Initially: 3×/week, each exercise consisting of a series of five repetitions Subsequently: 5×/week, 1×/day, with 10–15 repetitions, for 4 weeks

### Risk of study bias

**FIGURE 2:**
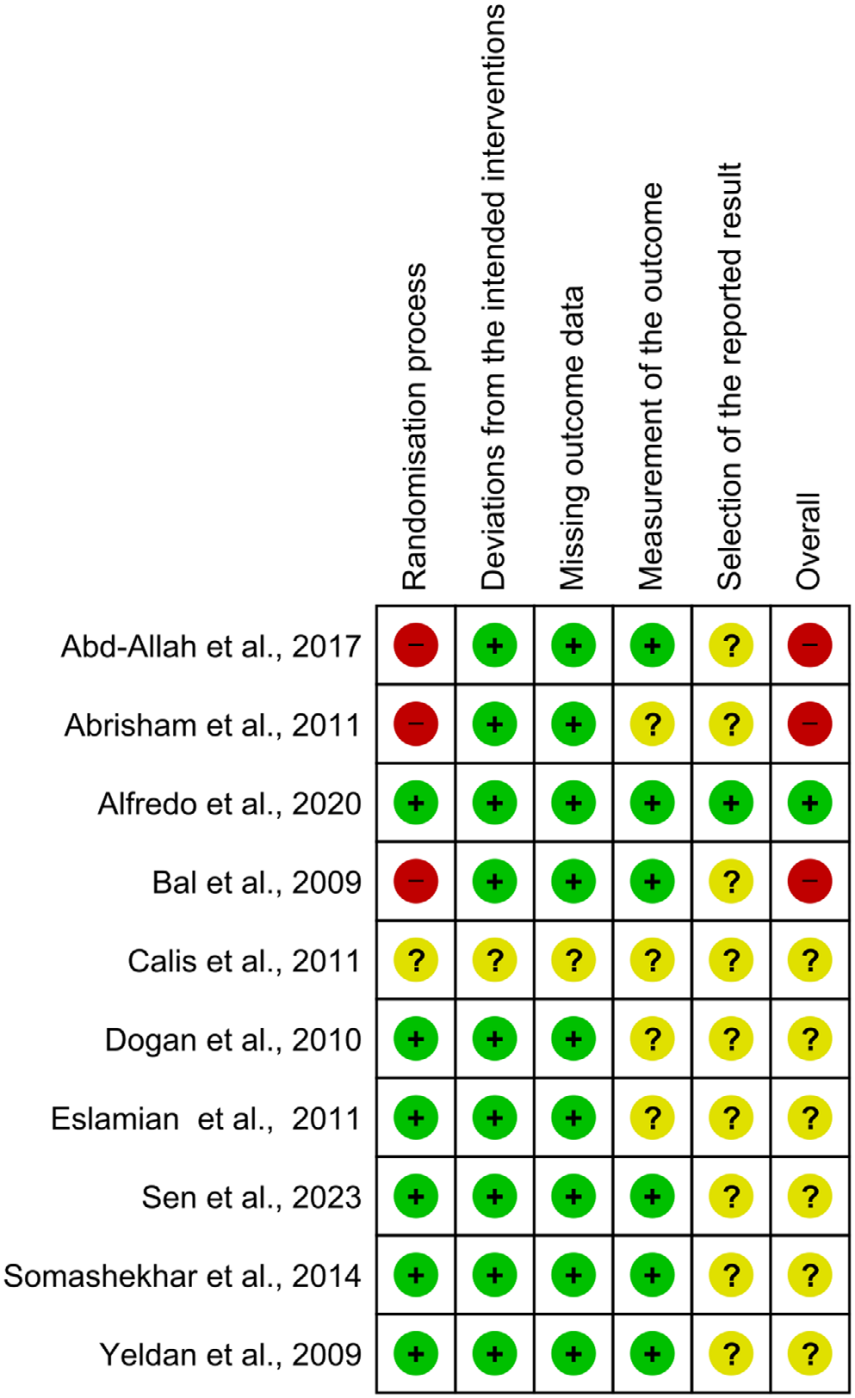
Risk of bias summary.

**FIGURE 3:**
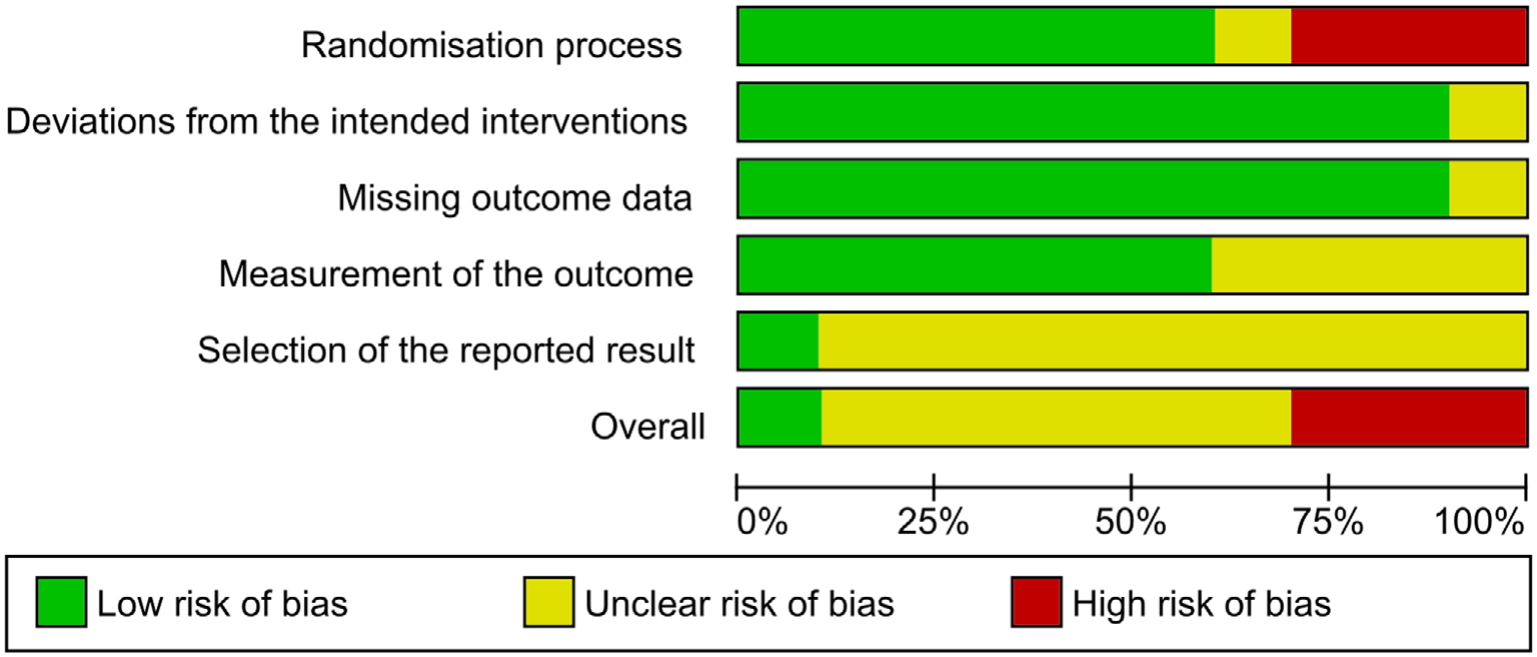
Risk of bias graph.

The individual risk of bias of the studies was assessed using the Cochrane tool, RoB2. Based on the analysis, one study was classified as low risk of bias.^31^ Another 6 studies had some concerns about risk of bias.^21^, ^23^, ^25^, ^26^, ^28^, ^30^ Finally, the studies by Abd‐Allah et al.,^29^ Abrisham et al.^27^ and Calis et al.^25^ were classified as having a high risk of bias. The domain with a high risk of bias was domain 1, the randomization process. Domain 5, the selection of reported results, had some concerns about risk of bias. The other domains, 2 through 4, had a low risk of bias (Figure 2). The review authors' judgments on each risk of bias item are presented in percentages for all of the studies included in Figure 3.

### Meta‐analysis

The outcomes evaluated in this review were meta‐analyzed. The primary outcome of pain and secondary outcomes of range of motion and functionality were analyzed.

#### Pain intensity

A total of ten studies^21^, ^22^, ^23^, ^25^, ^26^, ^27^, ^28^, ^29^, ^30^, ^31^ were included in the meta‐analysis of pain intensity, with a total of 523 individuals. The higher the score, the worse the pain intensity. In the original result of the meta‐analysis, before the sensitivity analysis, the pooled effect estimate showed no significant improvement in pain in the PBM group compared to the control [SMD = −0.27, 95% CI (−0.74, 0.19), *I* ^2^ 85%, *p* = 0.25].

**FIGURE 4:**
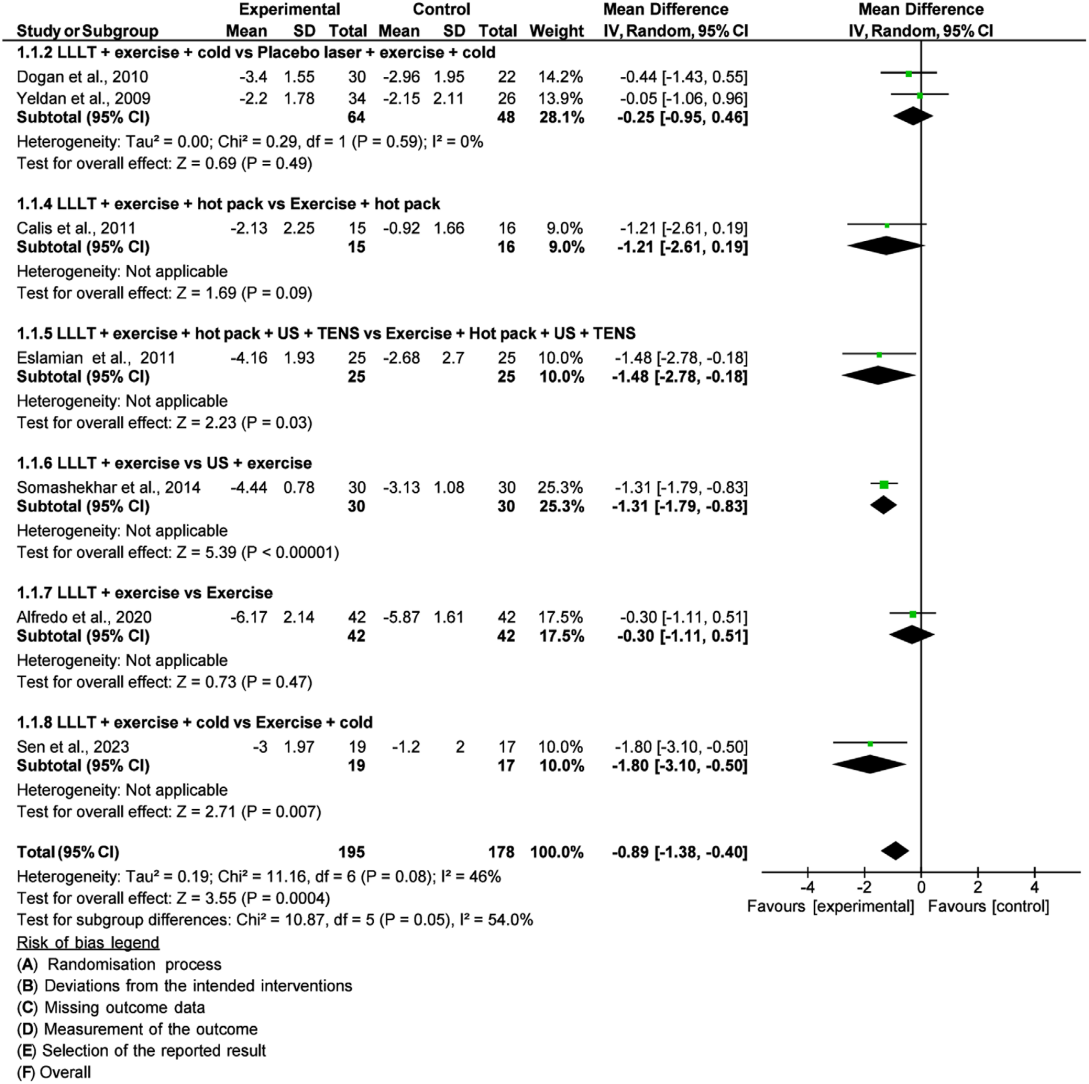
Pain intensity.

After the sensitivity analysis, with the removal of the three studies^22^, ^27^, ^29^ with a high risk of bias, the result of the meta‐analysis changed the estimate of the total effect [MD = −0.89, 95% CI (−1.38, −0.40), *I* ^2^ 46%, *p* = 0.0004]. For the remaining studies, the sample was 373 individuals (Figure 4).

#### Pain intensity at rest, activity, and at night

The studies that analyzed pain at specific times were those by Alfredo et al.,^31^ Calis et al.,^25^ Bal et al.,^22^ Sen et al.,^26^ and Yeldan et al.^23^ with a total of 251 subjects. Subgroup analysis was carried out to assess pain intensity in different circumstances. To do this, the results were stratified, generating three subgroups: pain intensity at rest, activity, and at night.

Pain intensity at rest ([SMD = 0.10; 95% CI (−0.50, 0.69) *p* = 76], *I* ^2^ 77%) and during activity ([SMD = −0.34; 95% CI (−0.71, 0.02) *p* = 0.07], *I* ^2^ 40%) was measured in four studies.^23^, ^25^, ^26^, ^31^ Pain intensity during the night ([SMD = −0.20; 95% CI (−0.52, 0.13) *p* = 0.24], *I* ^2^ 9%) was measured in another four studies.^22^, ^23^, ^25^, ^26^

**FIGURE 5:**
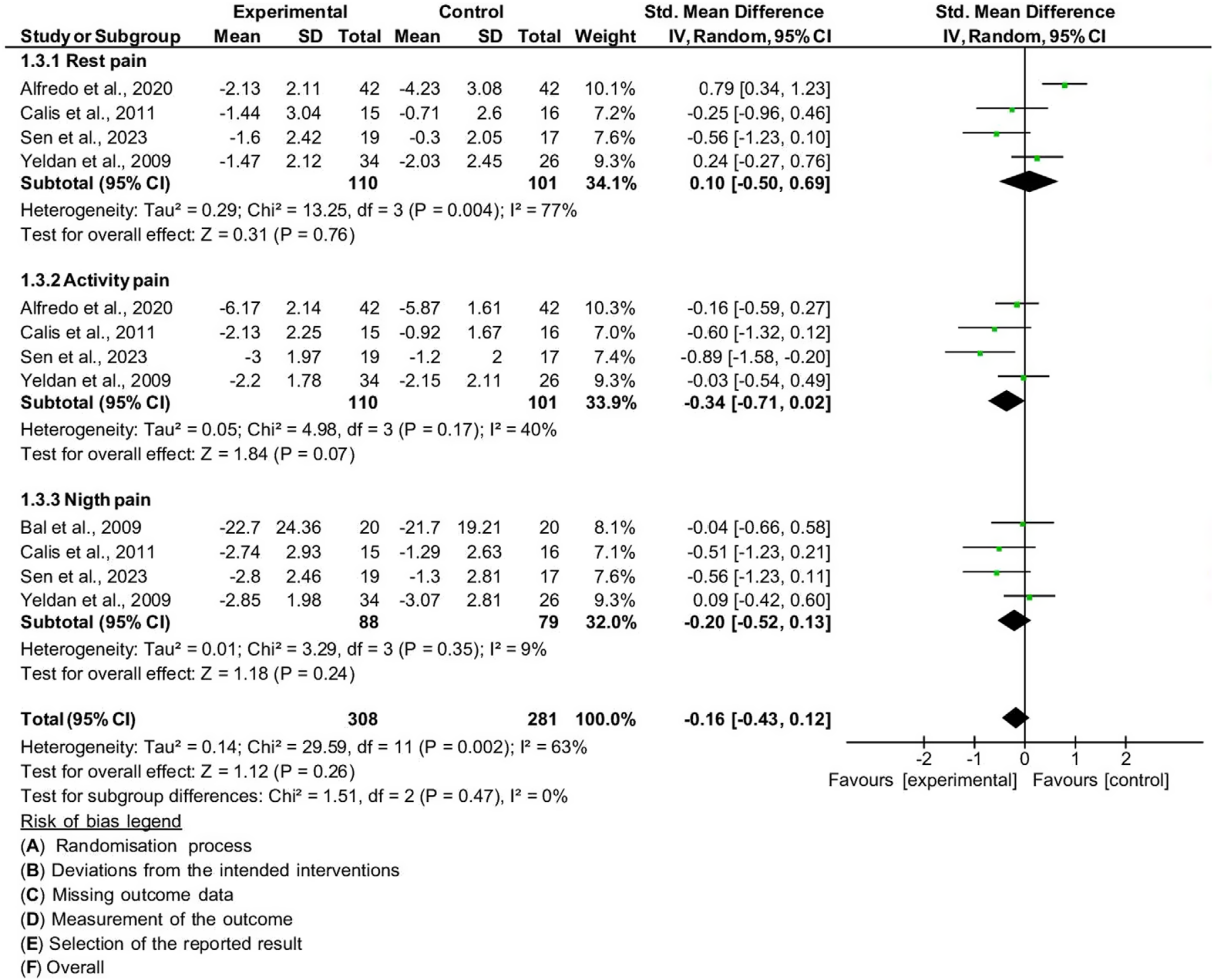
Pain Intensity – rest, activity, and night.

The 3 different times of assessment of pain intensity: at rest, activity, and at night were not statistically significant and likewise the combined effect size [SMD = −0.16; 95% CI (−0.43, 0.12) *p* = 0.26], according to the test of subgroup differences with substantial heterogeneity (*I* ^2^ = 63%) Figure 5. There was no improvement in pain intensity in any of the groups. After the sensitivity analysis with the removal of a study with a high risk of bias,^22^, ^27^, ^28^, ^31^ there was no change in the combined effect estimate [MD = −0.17, 95% CI (−0.47, 0.13), *I* ^2^ 66%, *p* = 0.26].

#### Range of motion

**FIGURE 6:**
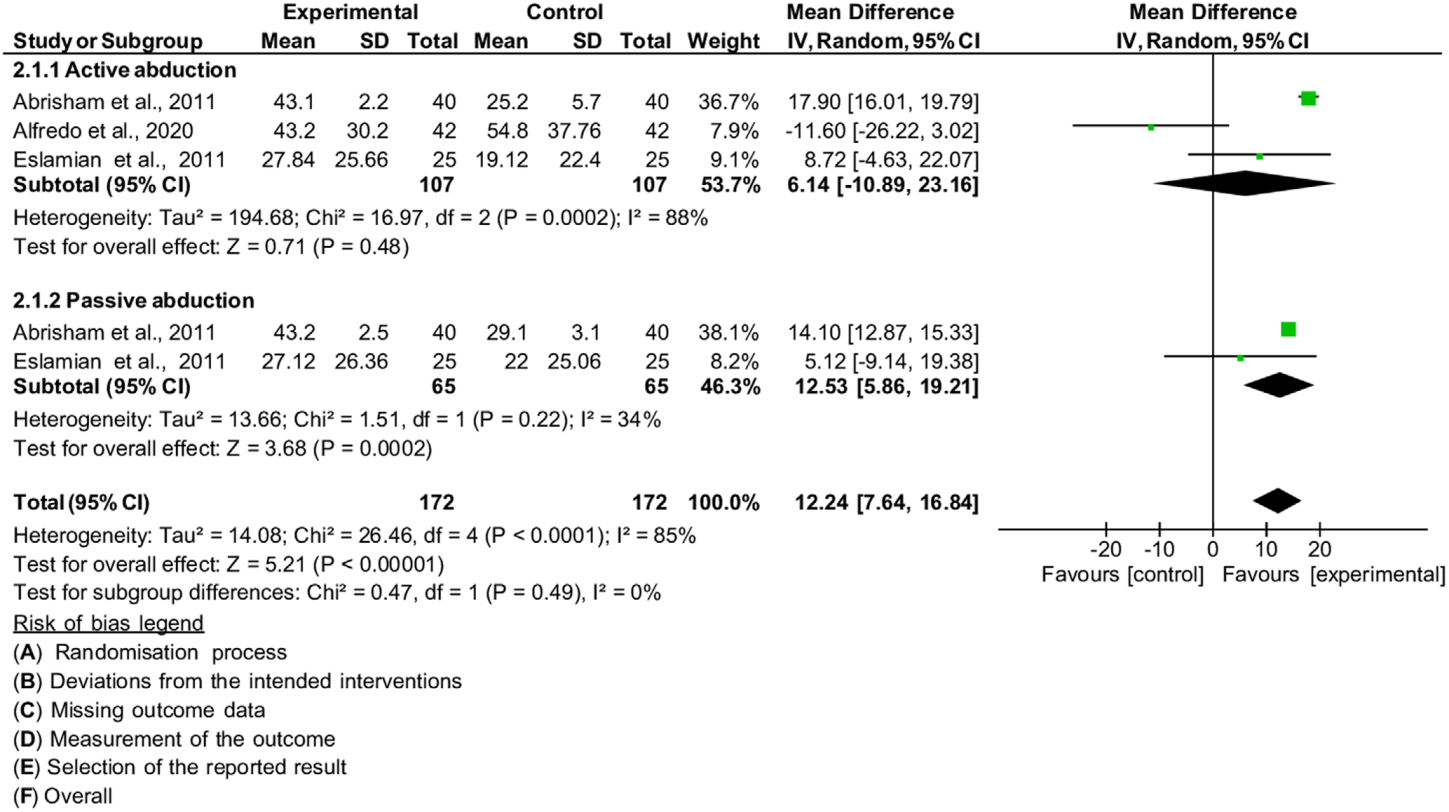
ROM – active and passive abduction.

Three studies^27^, ^28^, ^31^ were included in the meta‐analysis of active and passive abduction range of motion, with 214 individuals. The higher the score, the better the ROM. When PBM was compared to control, the subgroup analysis showed no improvement in active abduction range of motion in the PBM group [MD = 6.14, 95% CI (−10.89, 23.16), *I* ^2^ 88%, *p* = 0.48]. On the other hand, when PBM was compared to control, the subgroup analysis showed an increase in passive abduction range of motion in the PBM group [MD = 12.53, 95% CI (5.86, 19.21), *I* ^2^ 34%, *p* < 0.0002]. The result of the combined effect estimate showed a significant improvement in ROM in the PBM group compared to the control [MD = 12.24, 95% CI (7.64, 16.84), *I* ^2^ 85%, *p* < 0.00001], Figure 6.

#### Functionality

**FIGURE 7:**
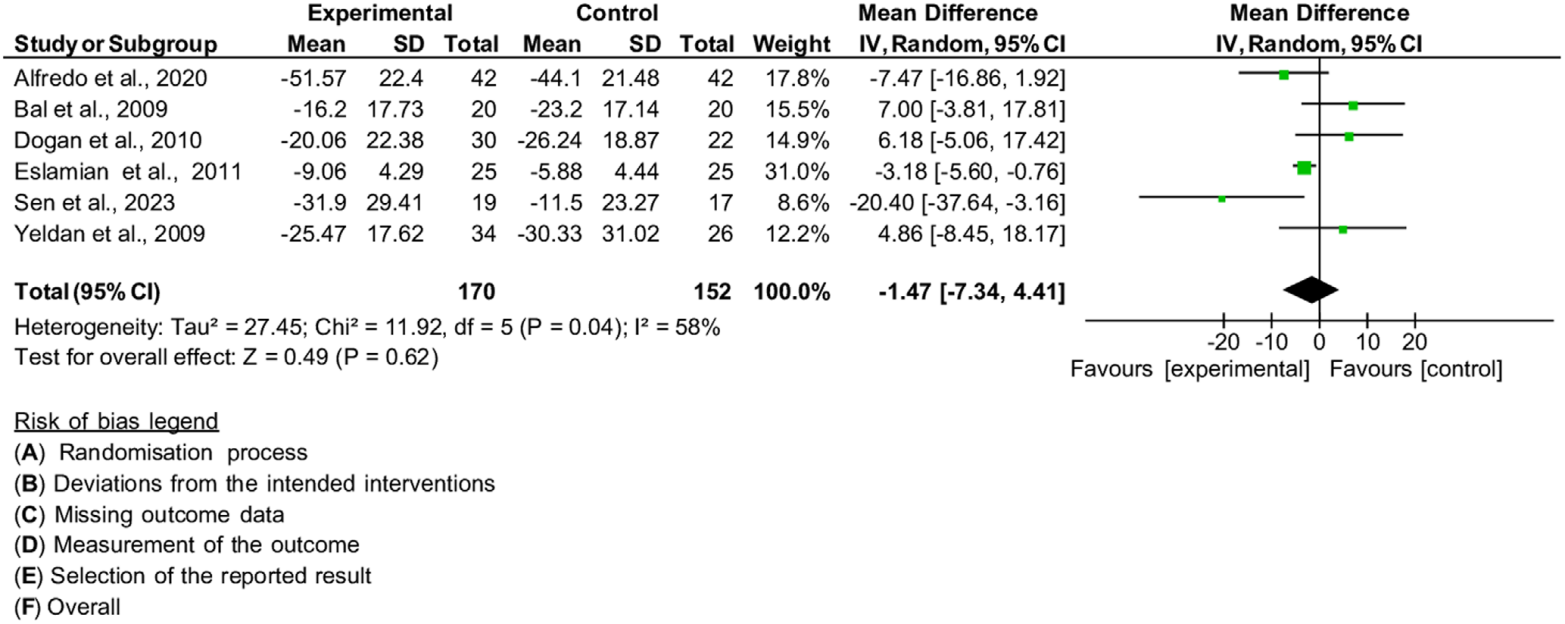
Functionality.

Six studies were included in the meta‐analysis in terms of functionality,^21^, ^22^, ^23^, ^26^, ^28^, ^31^ with the SPADI and Shoulder Disability Questionnaire (SDQ) instruments. In both instruments, the higher the score, the worse the functional status limitation. With the participation of 322 individuals, the result of the combined effect estimate showed no significant improvement in functionality in the PBM group compared to the control [MD = −1.47, 95% CI (−7.34, 4.41), *I* ^2^ 58%, *p* = 0.62], Figure 7. After the sensitivity analysis, even with the withdrawal of a study with a high risk of bias,^22^ there was no change in the combined effect estimate [MD = −3.00, 95% CI (−9.12, 3.11), *I* ^2^ 54%, *p* = 0.34].

### Risk of publication bias

**FIGURE 8:**
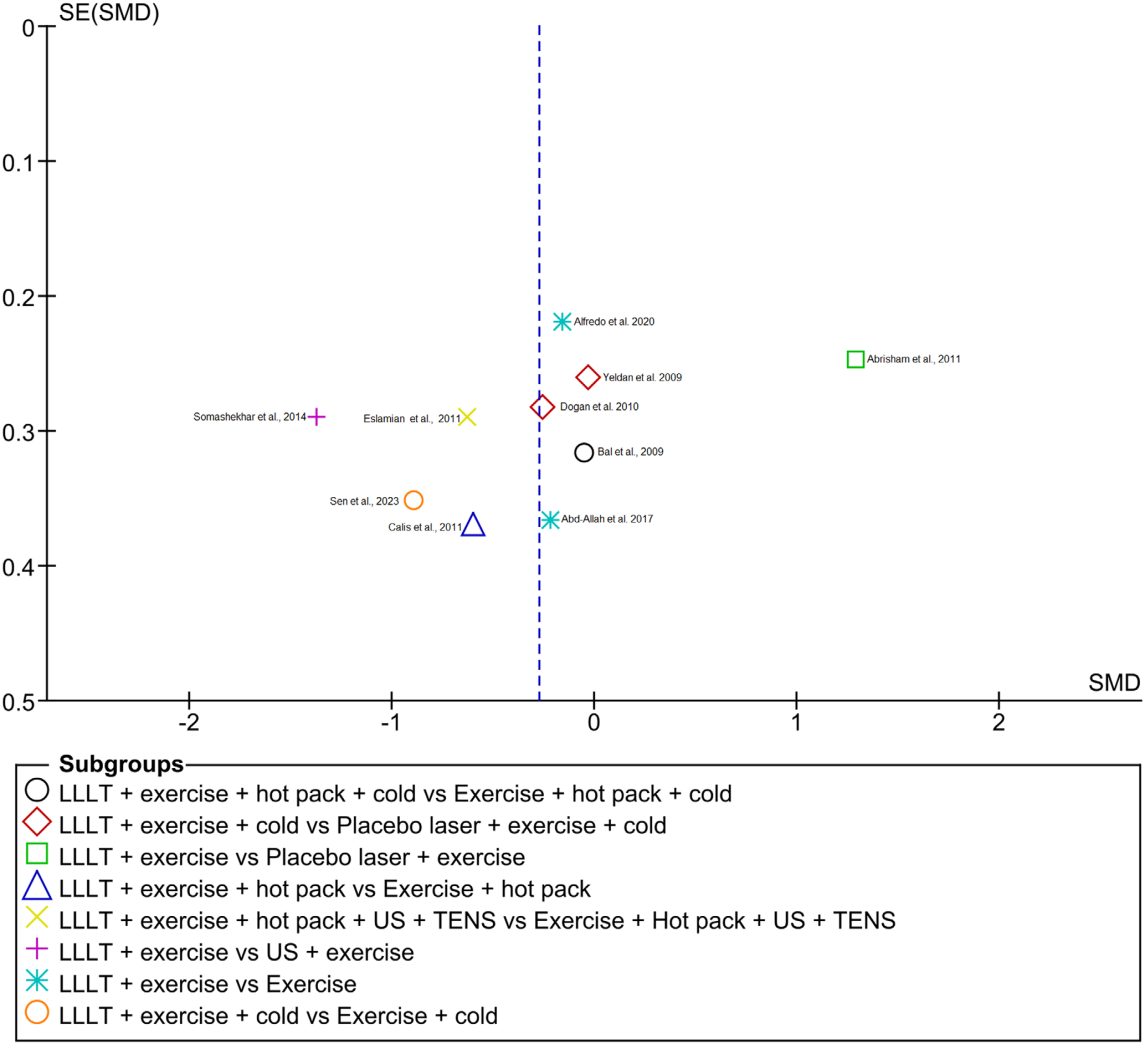
Risk of publication bias.

The funnel plot reflects different subgroups that were analyzed in the meta‐analysis, such as “LLLT + exercise + hot pack + cold vs. Exercise + hot pack + cold,” among other groups of the 10 included studies^21^, ^22^, ^23^, ^25^, ^26^, ^27^, ^28^, ^29^, ^30^, ^31^ (Figure 8). The combined analysis of the funnel plot and the meta‐analysis indicates that, although there is no clear evidence of publication bias, the high heterogeneity may be contributing to some variation in the results.

## DISCUSSION

This review aimed to present the effects of PBM in individuals with shoulder impingement syndrome and to present parameters of use. Ten randomized clinical trials (RCTs) that met the inclusion criteria for the use of PBM associated with physical exercise in the treatment of shoulder impingement syndrome were included.

PBM has shown itself to be a possible conservative therapeutic resource, as it has anti‐inflammatory effects, such as reducing the production of prostaglandin E2, inhibiting the activity of metalloproteinases, and accelerating mediators favorable to repair, as well as important analgesic effects. PBM has shown positive interaction with physical exercise. Another great advantage is the virtual absence of side effects. There are reports of no effects, which can be explained by the wide variety of dosimetric parameters.^32^, ^33^, ^34^, ^35^, ^36^, ^37^

In a systematic review published in 2016, Page et al.^38^ presented the use of electrothermophototherapeutic resources in rotator cuff diseases, citing that the use of low‐power laser, despite low evidence, was a modality that could be useful in these cases. The recent systematic review by Castaldo et al.,^39^ with a similar theme to the present study, showed that PBM was beneficial in reducing pain and increasing shoulder ROM. However, the authors did not analyze the risk of bias but rather analyzed the methodological quality using the Jadad scale, and probably due to the smaller number of databases searched, they did not find a number of similar publications to carry out a meta‐analysis.

In the present study, the findings are discussed in terms of pain intensity, range of motion, and shoulder functionality. PBM can produce alterations in the transmission of the nociceptive signal, with a reduction in the firing of the Aδ and C afferents,^40^, ^41^ reducing inflammatory mediators and enhancing repair,^32^, ^33^ thus creating the conditions for functional and ROM gains to occur with physical exercise; although in the present study, no improvement in functionality was observed.

With regard to the risk of bias, it was observed that only one study presented a low risk, six studies presented risk with some concerns, and three presented a high risk of general bias. Domain 1 (randomization process) was the most critical point due to the lack of concealment of the sequence of assignment of participants to the intervention. The heterogeneity of the parameters used in the included studies and the varying methods of application, together with the differences in treatment regimes, contribute to the difficulties in gathering information to allow definitive statements to be made about the use of PBM associated with exercise in shoulder impingement syndrome. Thus, the diversity of instruments used to measure outcomes, as well as the diversity of PBM parameters presented in tabular form, such as frequency and length of treatment, can be an important limitation, as it can limit the number of studies to be meta‐analyzed, especially when the interpretation of the Likert scale is different between them.

The following are some of the limitations found in this review. The intensity of pain, the main outcome of this review, was measured by all the studies, but only five studies reported whether the intensity of the pain declared referred to rest, activity, or during the night. The meta‐analysis therefore considered pain intensity during activity.^25^, ^26^, ^31^ In the study by Bal et al.,^22^ the analysis was of pain during the night, while the other studies did not report when pain was measured. This lack of information should be considered a limitation of this study. Similarly, ROM was reported in eight studies, but once again only three reported whether the movement was performed actively or passively.^27^, ^28^, ^31^

The high level of heterogeneity between the studies suggests that the aggregate results should be interpreted with caution. The significant differences between subgroups indicate that specific factors may be influencing the results, and these should be considered when interpreting the overall results of the meta‐analysis. This body of information suggests that although the overall effect is not significant, as seen in the pain intensity outcome before the sensitivity analysis, there is substantial variation in the effects observed between the studies, which may be influenced by different characteristics of the studies or interventions.

Meta‐analysis by subgroups was used to explore how different study characteristics might influence the observed effect. The overall analysis involved a larger sample size than any individual subgroup, increasing the statistical power to detect differences. In the present review, the sample size ranged from 17 to 42 individuals per group. In this way, small samples in subgroups may not have enough power to detect smaller effects, while the overall analysis, with a larger number of participants, may reveal a significant effect. As was the case with the range of motion (ROM) subgroup meta‐analysis, active abduction movement was not significant in relation to the control, but passive abduction was. The result of the combined effect estimate showed a significant improvement in ROM in the PBM group compared to the control.

The combined analysis of the funnel plot and the meta‐analysis indicates that, although there is no clear evidence of publication bias, the high heterogeneity may be contributing to some variation in the results. This should be taken into account when interpreting the conclusions of the review. It should be borne in mind that future RCTs should better address the randomization process, since it was the most critical point observed due to the lack of concealment of the sequence of assignment of participants to the intervention.

PBM is a useful resource, when combined with physical exercise, for reducing pain and gaining ROM in individuals with shoulder impingement syndrome. With regard to future trials, the most critical point in terms of risk of bias would be the randomization process, mainly due to the importance of adequate concealment of the sequence in which participants will be allocated to the intervention. It is therefore necessary to improve this area in order to guarantee the integrity of the results and reduce possible biases.

## Supporting information


Appendix S1

## Data Availability

Data sharing is not applicable to this article as no new data were created or analyzed in this study.
