# Supplementary material for: Photobiomodulation associated with physical exercise in shoulder impingement syndrome. Systematic review with meta‐analysis
Source: Photochem Photobiol. 2025 May 14;102(1):220–36. doi: 10.1111/php.14113 (PMC12807334; doi:10.1111/php.14113)
Supplement: Supplementary file 1 — Appendix S1 [file PHP-102-220-s001.docx]

SUPPLEMENTARY MATERIALS

There is a table as a supplementary document in the form of an appendix.

**Appendix 1** Search strategy in each database

| **Databases** | June/11/24 |
| --- | --- |
| **PubMed** | (((((("Rotator Cuff"[Mesh] ) OR ("Rotator Cuff") OR "Rotator Cuff Tear Arthropathy"[Mesh]) OR ("Rotator Cuff Tear Arthropathy") OR "Rotator Cuff Injuries"[Mesh] ) OR ("Rotator Cuff Injuries") OR "Shoulder Impingement Syndrome"[Mesh]) OR ("Shoulder Impingement Syndrome")) OR ("Shoulder Impingement Syndrome"[Title/Abstract]) OR "Milwaukee Shoulder" OR "Milwaukee Shoulder Syndrome" OR "Cuff Tear Arthropathy" OR "Rotator Cuff Injury" OR "Rotator Cuff Tears" OR "Rotator Cuff Tear" OR "Rotator Cuff Tendinosis" OR "Rotator Cuff Tendinoses" OR "Rotator Cuff Tendinitis" OR "Rotator Cuff Tendinitides" OR "Glenoid Labral Tears" OR "Glenoid Labral Tear" OR "Shoulder Impingement Syndromes" OR "Rotator Cuff Impingement" OR "Rotator Cuff Impingements" OR "Shoulder Impingement" OR "Shoulder Impingements" OR "Rotator Cuff Impingement Syndrome" OR "Coracohumeral Impingement Syndrome" OR "Coracohumeral Impingement Syndromes" OR "Coracoid Impingement Syndrome" OR "Coracoid Impingement Syndromes" OR "Coracohumeral Impingement" OR "Coracohumeral Impingements" OR "Subacromial Impingement Syndrome" OR "Subacromial Impingement Syndromes" OR "Outlet Impingement Syndrome" OR "Outlet Impingement Syndromes" OR "Outlet Impingement" OR "Outlet Impingements" OR "Internal Impingement Syndrome" OR "Internal Impingement Syndromes" OR "Posterosuperior Glenoid Impingement" OR "Posterosuperior Glenoid Impingements") AND (((((((((((("Low-Level Light Therapy"[Mesh] OR "Low-Level Light Therapy" ) OR ("Low-Level Light Therapy")) OR ("Low-Level Light Therapy"[Title/Abstract]) OR "Laser Therapy"[Mesh] ) OR ("Laser Therapy"[Title/Abstract])) OR ("Laser Therapy") OR "Low Level Light Therapy" OR "Low-Level Light Therapies" OR "Photobiomodulation Therapy" OR "Photobiomodulation Therapies" OR "LLLT" OR "Low-Level Laser Therapies" OR "Low-Power Laser Therapy" OR "Low Power Laser Therapy" OR "Low-Power Laser Therapies" OR "Low-Power Laser Irradiation" OR "Low Power Laser Irradiation" OR "Laser Biostimulation" OR "Laser Phototherapy" OR "Photostimulation" OR "Low Intensity Laser Therapy" OR "Laser Therapies, Low Level"[Mesh] OR "Laser Therapies, Low Level" OR "Laser Therapies, Low Power"[Mesh]) OR ("Laser Therapies, Low Power") OR "Laser Therapies, Low Power" OR "Laser Therapy, Low Level" OR "Laser Therapy, Low Level" OR "Laser Therapy, Low Power" [Mesh] ) OR ("Laser Therapy, Low Power") OR "Low Level Laser Therapies" [Mesh] ) OR ("Low Level Laser Therapies") OR "Low Level Laser Therapy" [Mesh] ) OR ("Low Level Laser Therapy") OR "Low Power Laser Therapies" [Mesh]) OR ("Low Power Laser Therapies") OR "Low Power Laser Therapy" [Mesh] ) OR ("Low Power Laser Therapy"))) |
| **Web of Science** | ("Rotator Cuff" OR "Rotator Cuff Tear Arthropathy" OR "Rotator Cuff Injuries" OR "Shoulder Impingement Syndrome" OR "Milwaukee Shoulder" OR "Milwaukee Shoulder Syndrome" OR "Cuff Tear Arthropathy" OR "Rotator Cuff Injury" OR "Rotator Cuff Tears" OR "Rotator Cuff Tear" OR "Rotator Cuff Tendinosis" OR "Rotator Cuff Tendinoses" OR "Rotator Cuff Tendinitis" OR "Rotator Cuff Tendinitides" OR "Glenoid Labral Tears" OR "Glenoid Labral Tear" OR "Shoulder Impingement Syndromes" OR "Rotator Cuff Impingement" OR "Rotator Cuff Impingements" OR "Shoulder Impingement" OR "Shoulder Impingements" OR "Rotator Cuff Impingement Syndrome" OR "Coracohumeral Impingement Syndrome" OR "Coracohumeral Impingement Syndromes" OR "Coracoid Impingement Syndrome" OR "Coracoid Impingement Syndromes" OR "Coracohumeral Impingement" OR "Coracohumeral Impingements" OR "Subacromial Impingement Syndrome" OR "Subacromial Impingement Syndromes" OR "Outlet Impingement Syndrome" OR "Outlet Impingement Syndromes" OR "Outlet Impingement" OR "Outlet Impingements" OR "Internal Impingement Syndrome" OR "Internal Impingement Syndromes" OR "Posterosuperior Glenoid Impingement" OR "Posterosuperior Glenoid Impingements") (Tópico) and ("Low-Level Light Therapy" OR "Laser Therapy" OR "Low Level Light Therapy" OR "Low-Level Light Therapies" OR "Photobiomodulation Therapy" OR "Photobiomodulation Therapies" OR "LLLT" OR "Low-Level Laser Therapies" OR "Low-Power Laser Therapy" OR "Low Power Laser Therapy" OR "Low-Power Laser Therapies" OR "Low-Power Laser Irradiation" OR "Low Power Laser Irradiation" OR "Laser Biostimulation" OR "Laser Phototherapy" OR "Photostimulation" OR "Low Intensity Laser Therapy" OR "Laser Therapies, Low Level" OR "Laser Therapies, Low Power" OR "Laser Therapy, Low Level" OR "Laser Therapy, Low Power" OR "Low Level Laser Therapies" OR "Low Level Laser Therapy" OR "Low Power Laser Therapies" OR "Low Power Laser Therapy") (Tópico) |
| **Scopus** | TITLE-ABS-KEY ( "Rotator Cuff" OR "Rotator Cuff Tear Arthropathy" OR "Rotator Cuff Injuries" OR "Shoulder Impingement Syndrome" OR "Milwaukee Shoulder" OR "Milwaukee Shoulder Syndrome" OR "Cuff Tear Arthropathy" OR "Rotator Cuff Injury" OR "Rotator Cuff Tears" OR "Rotator Cuff Tear" OR "Rotator Cuff Tendinosis" OR "Rotator Cuff Tendinoses" OR "Rotator Cuff Tendinitis" OR "Rotator Cuff Tendinitides" OR "Glenoid Labral Tears" OR "Glenoid Labral Tear" OR "Shoulder Impingement Syndromes" OR "Rotator Cuff Impingement" OR "Rotator Cuff Impingements" OR "Shoulder Impingement" OR "Shoulder Impingements" OR "Rotator Cuff Impingement Syndrome" OR "Coracohumeral Impingement Syndrome" OR "Coracohumeral Impingement Syndromes" OR "Coracoid Impingement Syndrome" OR "Coracoid Impingement Syndromes" OR "Coracohumeral Impingement" OR "Coracohumeral Impingements" OR "Subacromial Impingement Syndrome" OR "Subacromial Impingement Syndromes" OR "Outlet Impingement Syndrome" OR "Outlet Impingement Syndromes" OR "Outlet Impingement" OR "Outlet Impingements" OR "Internal Impingement Syndrome" OR "Internal Impingement Syndromes" OR "Posterosuperior Glenoid Impingement" OR "Posterosuperior Glenoid Impingements" ) AND TITLE-ABS-KEY ( "Low-Level Light Therapy" OR "Laser Therapy" OR "Low Level Light Therapy" OR "Low-Level Light Therapies" OR "Photobiomodulation Therapy" OR "Photobiomodulation Therapies" OR "LLLT" OR "Low-Level Laser Therapies" OR "Low-Power Laser Therapy" OR "Low Power Laser Therapy" OR "Low-Power Laser Therapies" OR "Low-Power Laser Irradiation" OR "Low Power Laser Irradiation" OR "Laser Biostimulation" OR "Laser Phototherapy" OR "Photostimulation" OR "Low Intensity Laser Therapy" OR "Laser Therapies, Low Level" OR "Laser Therapies, Low Power" OR "Laser Therapy, Low Level" OR "Laser Therapy, Low Power" OR "Low Level Laser Therapies" OR "Low Level Laser Therapy" OR "Low Power Laser Therapies" OR "Low Power Laser Therapy" ) |
| **Embase** | ('rotator cuff'/exp OR 'rotator cuff' OR 'rotator cuff tear arthropathy'/exp OR 'rotator cuff tear arthropathy' OR 'rotator cuff injuries'/exp OR 'rotator cuff injuries' OR 'shoulder impingement syndrome'/exp OR 'shoulder impingement syndrome' OR 'milwaukee shoulder'/exp OR 'milwaukee shoulder' OR 'milwaukee shoulder syndrome'/exp OR 'milwaukee shoulder syndrome' OR 'cuff tear arthropathy'/exp OR 'cuff tear arthropathy' OR 'rotator cuff injury'/exp OR 'rotator cuff injury' OR 'rotator cuff tears'/exp OR 'rotator cuff tears' OR 'rotator cuff tear'/exp OR 'rotator cuff tear' OR 'rotator cuff tendinosis' OR 'rotator cuff tendinoses' OR 'rotator cuff tendinitis'/exp OR 'rotator cuff tendinitis' OR 'rotator cuff tendinitides' OR 'glenoid labral tears' OR 'glenoid labral tear' OR 'shoulder impingement syndromes' OR 'rotator cuff impingement'/exp OR 'rotator cuff impingement' OR 'rotator cuff impingements' OR 'shoulder impingement'/exp OR 'shoulder impingement' OR 'shoulder impingements' OR 'rotator cuff impingement syndrome' OR 'coracohumeral impingement syndrome' OR 'coracohumeral impingement syndromes' OR 'coracoid impingement syndrome' OR 'coracoid impingement syndromes' OR 'coracohumeral impingement' OR 'coracohumeral impingements' OR 'subacromial impingement syndrome'/exp OR 'subacromial impingement syndrome' OR 'subacromial impingement syndromes' OR 'outlet impingement syndrome' OR 'outlet impingement syndromes' OR 'outlet impingement' OR 'outlet impingements' OR 'internal impingement syndrome' OR 'internal impingement syndromes' OR 'posterosuperior glenoid impingement' OR 'posterosuperior glenoid impingements') AND ('low-level light therapy'/exp OR 'low-level light therapy' OR 'laser therapy'/exp OR 'laser therapy' OR 'low level light therapy'/exp OR 'low level light therapy' OR 'low-level light therapies' OR 'photobiomodulation therapy'/exp OR 'photobiomodulation therapy' OR 'photobiomodulation therapies' OR 'lllt' OR 'low-level laser therapies' OR 'low-power laser therapy'/exp OR 'low-power laser therapy' OR 'low-power laser therapies' OR 'low-power laser irradiation' OR 'low power laser irradiation'/exp OR 'low power laser irradiation' OR 'laser biostimulation'/exp OR 'laser biostimulation' OR 'laser phototherapy' OR 'photostimulation'/exp OR 'photostimulation' OR 'low intensity laser therapy'/exp OR 'low intensity laser therapy' OR 'laser therapies, low level' OR 'laser therapies, low power' OR 'laser therapy, low level'/exp OR 'laser therapy, low level' OR 'laser therapy, low power' OR 'low level laser therapies' OR 'low level laser therapy'/exp OR 'low level laser therapy' OR 'low power laser therapies' OR 'low power laser therapy'/exp OR 'low power laser therapy') |
| **Lilacs** | (("Rotator Cuff" OR "Rotator Cuff Tear Arthropathy" OR "Rotator Cuff Injuries" OR "Shoulder Impingement Syndrome" OR "Milwaukee Shoulder" OR "Milwaukee Shoulder Syndrome" OR "Cuff Tear Arthropathy" OR "Rotator Cuff Injury" OR "Rotator Cuff Tears" OR "Rotator Cuff Tear" OR "Rotator Cuff Tendinosis" OR "Rotator Cuff Tendinoses" OR "Rotator Cuff Tendinitis" OR "Rotator Cuff Tendinitides" OR "Glenoid Labral Tears" OR "Glenoid Labral Tear" OR "Shoulder Impingement Syndromes" OR "Rotator Cuff Impingement" OR "Rotator Cuff Impingements" OR "Shoulder Impingement" OR "Shoulder Impingements" OR "Rotator Cuff Impingement Syndrome" OR "Coracohumeral Impingement Syndrome" OR "Coracohumeral Impingement Syndromes" OR "Coracoid Impingement Syndrome" OR "Coracoid Impingement Syndromes" OR "Coracohumeral Impingement" OR "Coracohumeral Impingements" OR "Subacromial Impingement Syndrome" OR "Subacromial Impingement Syndromes" OR "Outlet Impingement Syndrome" OR "Outlet Impingement Syndromes" OR "Outlet Impingement" OR "Outlet Impingements" OR "Internal Impingement Syndrome" OR "Internal Impingement Syndromes" OR "Posterosuperior Glenoid Impingement" OR "Posterosuperior Glenoid Impingements" OR "Cuff, Rotator" OR "Manguito Rotador" OR "Bainha Rotadora" OR "Subacromial Impingement Syndrome" OR "Síndrome de Abducción Dolorosa del Hombro" OR "Síndrome de Pinzamiento del Hombro" OR "Síndrome del Supraespinoso" OR "Síndrome del Arco Doloroso" OR "Síndrome de Hombro Doloroso" OR "Síndrome del Hombro Doloroso" OR "Síndrome de Pinzamiento Subacromial" OR "Síndrome de Colisão do Ombro" OR "Síndrome de Impacto do Ombro" OR "Síndrome do Impacto do Ombro" OR "Síndrome de Pinçamento do Ombro" OR "Síndrome do Supraespinal" OR "Síndrome do Arco Doloroso" OR "Síndrome de Colisão Subacromial" OR "Síndrome de Pinçamento Subacromial") ) AND (("Low-Level Light Therapy" OR "Low Level Light Therapy" OR "Low-Level Light Therapies" OR "Photobiomodulation Therapy" OR "Photobiomodulation Therapies" OR "LLLT" OR "Low-Level Laser Therapies" OR "Low-Power Laser Therapy" OR "Low Power Laser Therapy" OR "Low-Power Laser Therapies" OR "Low-Level Laser Therapy" OR "Low Level Laser Therapy" OR "Low-Power Laser Irradiation" OR "Low Power Laser Irradiation" OR "Laser Biostimulation" OR "Laser Phototherapy" OR "Photostimulation" OR "Low Intensity Laser Therapy" OR "Terapia por Luz de Baja Intensidad" OR "Terapia por Láser de Baja Intensidad" OR "Irradiación por Láser de Bajo Poder" OR "Terapia por Láser de Bajo Nivel" OR "Terapia por Láser de Baja Potencia" OR "Bioestimulación por Láser" OR "Irradiación por Láser de Baja Potencia" OR "Terapia com Luz de Baixa Intensidade" OR "Terapia a Laser de Baixa Intensidade" OR "Irradiação a Laser de Baixa Intensidade" OR "Terapia a Laser de Baixa Potência" OR "Bioestimulação a Laser" OR "Irradiação a Laser de Baixa Potência") ) |
| **Cochrane** | Trials matching ("Rotator Cuff" OR "Rotator Cuff Tear Arthropathy" OR "Rotator Cuff Injuries" OR "Shoulder Impingement Syndrome" OR "Milwaukee Shoulder" OR "Milwaukee Shoulder Syndrome" OR "Cuff Tear Arthropathy" OR "Rotator Cuff Injury" OR "Rotator Cuff Tears" OR "Rotator Cuff Tear" OR "Rotator Cuff Tendinosis" OR "Rotator Cuff Tendinoses" OR "Rotator Cuff Tendinitis" OR "Rotator Cuff Tendinitides" OR "Glenoid Labral Tears" OR "Glenoid Labral Tear" OR "Shoulder Impingement Syndromes" OR "Rotator Cuff Impingement" OR "Rotator Cuff Impingements" OR "Shoulder Impingement" OR "Shoulder Impingements" OR "Rotator Cuff Impingement Syndrome" OR "Coracohumeral Impingement Syndrome" OR "Coracohumeral Impingement Syndromes" OR "Coracoid Impingement Syndrome" OR "Coracoid Impingement Syndromes" OR "Coracohumeral Impingement" OR "Coracohumeral Impingements" OR "Subacromial Impingement Syndrome" OR "Subacromial Impingement Syndromes" OR "Outlet Impingement Syndrome" OR "Outlet Impingement Syndromes" OR "Outlet Impingement" OR "Outlet Impingements" OR "Internal Impingement Syndrome" OR "Internal Impingement Syndromes" OR "Posterosuperior Glenoid Impingement" OR "Posterosuperior Glenoid Impingements") in Title Abstract Keyword AND ("Low-Level Light Therapy" OR "Laser Therapy" OR "Low Level Light Therapy" OR "Low-Level Light Therapies" OR "Photobiomodulation Therapy" OR "Photobiomodulation Therapies" OR "LLLT" OR "Low-Level Laser Therapies" OR "Low-Power Laser Therapy" OR "Low Power Laser Therapy" OR "Low-Power Laser Therapies" OR "Low-Power Laser Irradiation" OR "Low Power Laser Irradiation" OR "Laser Biostimulation" OR "Laser Phototherapy" OR "Photostimulation" OR "Low Intensity Laser Therapy" OR "Laser Therapies, Low Level" OR "Laser Therapies, Low Power" OR "Laser Therapy, Low Level" OR "Laser Therapy, Low Power" OR "Low Level Laser Therapies" OR "Low Level Laser Therapy" OR "Low Power Laser Therapies" OR "Low Power Laser Therapy") in Title Abstract Keyword - (Word variations have been searched) |
| **LIVIVO** | ("Rotator Cuff"[Mesh] OR "Rotator Cuff" OR "Rotator Cuff Tear Arthropathy"[Mesh] OR "Rotator Cuff Tear Arthropathy" "Rotator Cuff Injuries"[Mesh] OR "Rotator Cuff Injuries" OR "Shoulder Impingement Syndrome"[Mesh] OR "Shoulder Impingement Syndrome" OR "Milwaukee Shoulder" OR "Milwaukee Shoulder Syndrome" OR "Cuff Tear Arthropathy" OR "Rotator Cuff Injury" OR "Rotator Cuff Tears" OR "Rotator Cuff Tear" OR "Rotator Cuff Tendinosis" OR "Rotator Cuff Tendinoses" OR "Rotator Cuff Tendinitis" OR "Rotator Cuff Tendinitides" OR "Glenoid Labral Tears" OR "Glenoid Labral Tear" OR "Shoulder Impingement Syndromes" OR "Rotator Cuff Impingement" OR "Rotator Cuff Impingements" OR "Shoulder Impingement" OR "Shoulder Impingements" OR "Rotator Cuff Impingement Syndrome" OR "Coracohumeral Impingement Syndrome" OR "Coracohumeral Impingement Syndromes" OR "Coracoid Impingement Syndrome" OR "Coracoid Impingement Syndromes" OR "Coracohumeral Impingement" OR "Coracohumeral Impingements" OR "Subacromial Impingement Syndrome" OR "Subacromial Impingement Syndromes" OR "Outlet Impingement Syndrome" OR "Outlet Impingement Syndromes" OR "Outlet Impingement" OR "Outlet Impingements" OR "Internal Impingement Syndrome" OR "Internal Impingement Syndromes" OR "Posterosuperior Glenoid Impingement" OR "Posterosuperior Glenoid Impingements") AND ("Low-Level Light Therapy"[Mesh] OR "Low-Level Light Therapy" OR "Laser Therapy"[Mesh] OR "Laser Therapy" OR "Low Level Light Therapy" OR "Low-Level Light Therapies" OR "Photobiomodulation Therapy" OR "Photobiomodulation Therapies" OR "LLLT" OR "Low-Level Laser Therapies" OR "Low-Power Laser Therapy" OR "Low Power Laser Therapy" OR "Low-Power Laser Therapies" OR "Low-Power Laser Irradiation" OR "Low Power Laser Irradiation" OR "Laser Biostimulation" OR "Laser Phototherapy" OR "Photostimulation" OR "Low Intensity Laser Therapy" OR "Laser Therapies, Low Level"[Mesh] OR "Laser Therapies, Low Level" OR "Laser Therapies, Low Power"[Mesh] OR "Laser Therapies, Low Power" OR "Laser Therapy, Low Level" OR "Laser Therapy, Low Level" OR "Laser Therapy, Low Power" [Mesh] OR "Laser Therapy, Low Power" OR "Low Level Laser Therapies" [Mesh] OR "Low Level Laser Therapies" OR "Low Level Laser Therapy" [Mesh] OR "Low Level Laser Therapy" OR "Low Power Laser Therapies" [Mesh] OR "Low Power Laser Therapies" OR "Low Power Laser Therapy" [Mesh] OR "Low Power Laser Therapy") |
| **Google scholar** | (Shoulder Impingement Syndrome) AND (Low Level Light Therapy) |
| **Open Grey** | ("Rotator Cuff" OR "Rotator Cuff Tear Arthropathy" OR "Rotator Cuff Injuries" OR "Shoulder Impingement Syndrome" OR "Milwaukee Shoulder" OR "Milwaukee Shoulder Syndrome" OR "Cuff Tear Arthropathy" OR "Rotator Cuff Injury" OR "Rotator Cuff Tears" OR "Rotator Cuff Tear" OR "Rotator Cuff Tendinosis" OR "Rotator Cuff Tendinoses" OR "Rotator Cuff Tendinitis" OR "Rotator Cuff Tendinitides" OR "Glenoid Labral Tears" OR "Glenoid Labral Tear" OR "Shoulder Impingement Syndromes" OR "Rotator Cuff Impingement" OR "Rotator Cuff Impingements" OR "Shoulder Impingement" OR "Shoulder Impingements" OR "Rotator Cuff Impingement Syndrome" OR "Coracohumeral Impingement Syndrome" OR "Coracohumeral Impingement Syndromes" OR "Coracoid Impingement Syndrome" OR "Coracoid Impingement Syndromes" OR "Coracohumeral Impingement" OR "Coracohumeral Impingements" OR "Subacromial Impingement Syndrome" OR "Subacromial Impingement Syndromes" OR "Outlet Impingement Syndrome" OR "Outlet Impingement Syndromes" OR "Outlet Impingement" OR "Outlet Impingements" OR "Internal Impingement Syndrome" OR "Internal Impingement Syndromes" OR "Posterosuperior Glenoid Impingement" OR "Posterosuperior Glenoid Impingements") AND ("Low-Level Light Therapy" OR "Laser Therapy" OR "Low Level Light Therapy" OR "Low-Level Light Therapies" OR "Photobiomodulation Therapy" OR "Photobiomodulation Therapies" OR "LLLT" OR "Low-Level Laser Therapies" OR "Low-Power Laser Therapy" OR "Low Power Laser Therapy" OR "Low-Power Laser Therapies" OR "Low-Power Laser Irradiation" OR "Low Power Laser Irradiation" OR "Laser Biostimulation" OR "Laser Phototherapy" OR "Photostimulation" OR "Low Intensity Laser Therapy" OR "Laser Therapies, Low Level" OR "Laser Therapies, Low Power" OR "Laser Therapy, Low Level" OR "Laser Therapy, Low Power" OR "Low Level Laser Therapies" OR "Low Level Laser Therapy" OR "Low Power Laser Therapies" OR "Low Power Laser Therapy") |
| **Catálogo de Tese e dissertação da CAPES** | (Shoulder Impingement Syndrome) AND (Low Level Light Therapy) |
